# The Effect of Microwave Pretreatment on Micronutrient Contents, Oxidative Stability and Flavor Quality of Peanut Oil

**DOI:** 10.3390/molecules24010062

**Published:** 2018-12-25

**Authors:** Hui Hu, Hongzhi Liu, Aimin Shi, Li Liu, Marie Laure Fauconnier, Qiang Wang

**Affiliations:** 1 Institute of Food Science and Technology, Chinese Academy of Agricultural Sciences/Key Laboratory of Agro-Products Processing, Ministry of Agriculture, P.O. Box 5109, Beijing 100193, China; huhui@caas.cn (H.H.); lhz0416@126.com (H.L.); sam_0912@163.com (A.S.); liulicaas@126.com (L.L.); 2 Laboratory of General and Organic Chemistry, University of Liege, Gembloux Agro-Bio Tech, Passage des Déportés, 2-5030 Gembloux, Belgium; marie-laure.fauconnier@ulg.ac.be

**Keywords:** peanut, cold press, microwave pretreatment, Phytosterol, Tocopherol, oxidative stability, Pyrazines

## Abstract

The purpose of the present study is to investigate the changes in extraction yield, physicochemical properties, micronutrients content, oxidative stability and flavor quality of cold pressed peanut oil extracted from microwave (MW) treated seeds (0, 1, 2, 3, 4, 5 min, 700 W). The acid value and peroxide value of extracted oil from MW-treated peanuts were slightly increased but far below the limit in the Codex standard. Compared with the untreated sample, a significant (*p* < 0.05) increase in extraction yield (by 33.75%), free phytosterols content (by 32.83%), free tocopherols content (by 51.36%) and induction period (by 168.93%) of oil extracted from 5 min MW-treated peanut were observed. MW pretreatment formed pyrazines which contribute to improving the nutty and roasty flavor of oil. In conclusion, MW pretreatment is a feasible method to improve the oil extraction yield and obtain the cold pressed peanut oil with longer shelf life and better flavor.

## 1. Introduction

Peanut is one of the most important oil crops in the world and is also an important source of protein. The worldwide production of peanuts reached 43.98 million tons [1]. Oil and food production are the two main uses of peanuts [2]. In 2017/18, the worldwide production of peanut oil was 5.92 million tons, among which approximately 50% were produced in China [3].

Peanuts are a nutrient-dense food that is rich in unsaturated fatty acids, fiber, vitamins, minerals, and many other bioactive substances. Clinical trials have suggested that compared with participants who did not eat nuts, those who consumed peanuts seven or more times per week had a 20% lower death rate [4]. The total amount of unsaturated fatty acid is over 85% in peanut oil. The fatty acid profile of peanut oil resembles that of olive oil, which could reduce the risk of cardiovascular disease [2]. Peanut oil is also rich in sterols (900–4344 mg/kg) and tocopherols (137–934 mg/kg) which have effects in enhancing immunity, reducing the incidence of car-diovascular diseases, lowing serum cholesterol, preventing cancer, and improving the oxidative stability of oil [5,6,7].

There are two main types of industrial peanut oil-processing methods: high-temperature pressing and cold pressing. More than 90% of peanut oil production in China is performed by the traditional technique of high-temperature pressing [2]. The aromatic roasted peanut oil obtained by this method is more popular with consumers because of the strong typical flavor. However, the pretreatment of roasting and high-temperature pressing leads to the loss of micronutrients and poor oil stability. The peanut oil produced by cold pressing maintains the original quality of the peanut, and a peanut protein meal with low denaturation level could be further produced for food use. However, when compared with hot-temperature pressing, lower oil extraction yield and weaker roasted flavors of the oils were the main differences with cold-pressing. In order to reduce this, a new pretreatment is required to replace the traditional treatment before cold pressing.

In the last two decades, new technologies for improving oil recovery have attracted research attention. The feasibility of using microwaves (MW) before or during oil processing has been widely studied and has confirmed its efficiency on improving the extraction yield, nutritional value, physicochemical and sensorial properties of oil [8]. Higher oil extraction yield was observed with MW pretreatment on rapeseed, palm, soybean, rice bran, cottonseed, Moringa oleifera seeds, black seed, chia seed, and Chilean hazelnuts [9,10,11,12,13,14,15,16]. Three minutes of MW pretreatment on rapeseed with 9% moisture content increased oil extraction yield by 16-19%, and the damage to the lipoprotein membrane was distinctly seen in scanning electron micrographs, which improved the oil extraction efficiency [17]. The effect of MW treatment on the physicochemical quality of oil has been investigated in recent years. After 10 min MW pretreatment, the peroxide value (PV) of rapeseed increased from 0.99–1.14 meq O_2_/kg to 2.23–2.27 meq O_2_/kg, which is within the Codex Alimentarius limits (PV < 15 meq O_2_/kg) [18]. The acid value (AV) of extracted oil from MW-treated hazelnuts increased from 1.56 mg KOH/g oil to 1.83 mg KOH/g, which was attributed to triacylglycerols hydrolysis [9]. As a contrary result, MW pretreatment caused a significant decrease in the AV of black seed oil (4.85–3.03 mg KOH/g), which can be attributed to lipase inactivation due to the thermal pretreatment [15]. The effect of MW on micronutrients content and oxidative stability of oil has been reported in other research [17,19]. Rapeseed with moisture levels of 9–15% had individual and total tocopherols in the extracted oils which first increased, then decreased according to the period of microwave radiation. With 7 min MW treatment of rapeseed with 9% moisture, the phytosterols and polyphenols in extracted oil reached maximum values (922.48 mg/100 g and 96.91 TA.100 g), which were 18.04% and 176.88% higher, respectively, than the untreated sample. On the contrary, the reduction of tocopherol contents compared with the control group was observed in oil extracted from MW-treated chia seed [16]. The induction period of extracted oil increased from 7.46 h to 22.80 h due to the increased micronutrients with antioxidant activity [20]. The effect of MW treatment on the flavor quality of oil has been investigated. The pyrazine compounds in the rapeseed oil appeared after 6 min of microwave pretreatment, this may be the main reason for giving a pleasant roasting flavor when compared to crude oils [21].

Studies on the microwave treatment effect on the quality of peanut oil are still lacking. The object of this study was to investigate the changes in extraction yield, physicochemical properties, micronutrients content, oxidative stability, and flavor quality of peanut oil extracted by cold pressing after microwave pretreatment. The results of this study will be used to evaluate the feasibility of using microwaves pretreatment as an improvement method for cold-pressed peanut oil processing.

## 2. Results and Discussion

### 2.1. Effect of Microwave Pretreatment on Oil Extraction Yield

**Figure 1:**
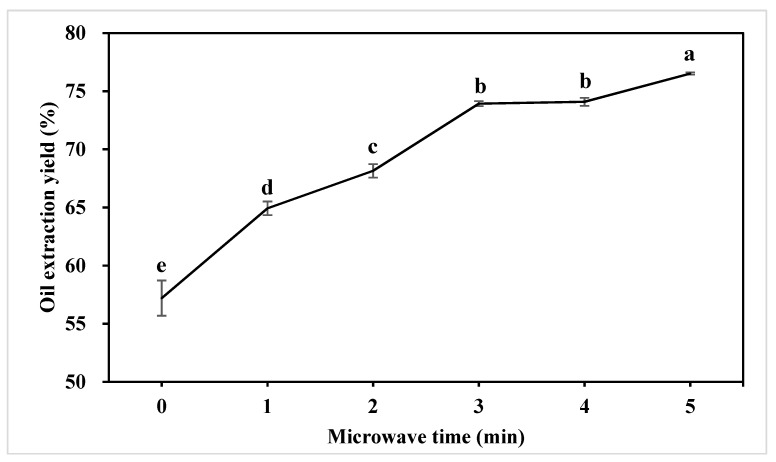
Effect of microwave pretreatment on the oil extraction yield. Different characters (a–e) on top of the line indicate significant (*p* < 0.05) differences among samples with different treatment times.

Oil extraction yield is one of the key indexes to evaluate the production efficiency of oil from oilseeds. MW treated and untreated peanut samples were cold-pressed to study the effect of MW pretreatment on oil extraction yield. The initial oil content of peanut was 48.90 ± 0.30%. The oil extraction yield of untreated peanut was 57.21 ± 1.51%. As can be seen from Figure 1, the increasing microwave pretreatment time significantly (*p* < 0.05) increased the oil extraction yield. For the 1, 2, 3 min MW-treated peanut, the oil extraction yield increased to 64.93 ± 0.58%, 68.16 ± 0.59% and 73.93 ± 0.21%, respectively. Five minutes of MW treatment reached the maximum oil extraction yield (76.52 ± 0.10%), which was 33.75% greater compared to the control. The results are consistent with previous studies of the MW pretreatment effect on the extraction yield of rapeseed oil, palm oil, soybean oil, rice bran oil, cottonseed oil, and Chilean hazelnuts oil [9,10,11,12,13,14,20,22,23]. With 2–10 min MW pretreatment on 10.5% moisture rapeseed, the oil extraction efficiency by cold pressing gradually increased from 39.21% to 53.73% [23]. The oil extraction efficiency by solvent extraction increased with longer irradiation time (up to 3.5 min), and the optimum extraction conditions resulted in a cottonseed oil extraction efficiency of 32.6% [13]. MW pretreatment to oilseeds improved the oil yield of both extraction methods (mechanical pressing and solvent extraction). Microscopic studies on the MW-treated oilseed structure changes showed the MW resulted in protein denaturation which could cause damages in the lipoprotein membrane surrounding individual lipid bodies. These changes promote the passage of oil from the cell membrane, which leads to improving the oil release efficiency during the extraction procedure [9,14,22].

### 2.2. Effect of Microwave Pretreatment on Physicochemical Properties of Oil

Color plays a very important role in the sensory properties of oil. As shown in Table 1, the MW treatment significantly (*p* < 0.05) affected the Lovibond color of extracted peanut oils. With the increasing MW treatment time, the color of extracted oil gradually changed from light yellow (R 0.00/Y 1.20, Control) to light brown (R 0.40/Y 3.47, 5 min MW treatment). Darkening of oils produced from roasted seeds was also reported by other researchers [18,24,25]. The changes in color may be due to the formation of phospholipid non-enzymatic browning products. MW pretreatment not only resulted in the darkening of oil but also led to an increased browning index [19], which was consistent with the result of oil extracted from roasted pine nut [26]. High correlation between the browning reaction markers (absorbance, fluorescence, and pyrrolyzed phospholipid content) could indicate that the brown compounds in the roasted seed oil are due to the occurrence of a Maillard type browning reaction of phospholipids [27].

Acid Value (AV) is a measure of the free fatty acids concentration in oil, which could be used to evaluate the freshness of oilseed, crude oil and product oil. As can be seen from Table 1, the AV of extracted oil from untreated peanut was 0.31 ± 0.02 mg KOH/g oil. As the peanuts were being MW treated, the AV of extracted oil was significantly (*p* < 0.05) increased. With 5 min MW treatment, the extracted oil reached the maximum AV (0.47 ± 0.00 mg KOH/g oil). Although it was 0.16 mg KOH/g higher than the control, it was still far below the limit in the Codex standard which allows for the presence of AV up to 4 mg KOH/g in cold-pressed and virgin oils. With the MW pretreatment, the decreased triacylglycerols (TAG) and increased free fatty acids were observed [24]. It is indicated that the increasing AV of extracted oil from MW-treated peanut may be due to the hydrolysis of TAG, as reported by other researchers [9,25,28].

Peroxide value (PV) measures the quantity of peroxides in the oil, which serves as an indicator index of the primary oxidation product formation. Very low PV (0.54 ± 0.08 meq O_2_/kg oil) was determined in the oil extracted from the control sample (Table 1). The PV of the oil significantly (*p* < 0.05) increased with the increasing MW treatment time. The peanuts were MW treated for 5 min and the extracted oil reached the maximum PV (5.30 ± 0.08 meq O_2_/kg oil), which was also within the Codex standard of cold-pressed and virgin oils (PV < 15 meq O_2_/kg oil). Similar results were observed in the MW pretreatment of rapeseed, Chilean hazelnuts and sunflower seed [9,18,25]. The effect of MW pretreatment on PV changes of extracted oil during the storage has also been studied. Even the initial PV of extracted oil from MW-treated rapeseed (0.85 mmol/kg) was higher than the initial PV of oil from untreated rapeseed (0.70 mmol/kg), but when stored for 32d, the PV of MW-treated oil (12.83 mmol/kg) was lower than the PV of untreated oil (20.22 mmol/kg) [29]. It can be inferred that MW pretreatment samples produced lower secondary oxidation products than the untreated samples. This result was consistent with previous research [27,28].

### 2.3. Effect of Microwave Pretreatment on the Free Phytosterols and Tocopherols Content of Oil

Phytosterols are not only generally recognized as providing significant lowering of serum low-density lipoprotein (LDL) cholesterol in humans, but has also shown protection against various chronic ailments like cardiovascular disease, hepatoprotective, diabetes, and cancer [6,7]. The main phytosterol sources are vegetable oils. The free-form sterols have higher efficacies (in human subjects) than sterol esters [6]. The effect of MW pretreatment on free phytosterols content in extracted oil is shown in Table 2. The free phytosterols content of oil from untreated peanut was 273.55 ± 2.51 mg/100 g. The content of free phytosterols significantly (*p* < 0.05) increased with increasing MW treatment time. With 5 min of MW treatment, the free phytosterols in the extracted oil reached the maximum content (363.35 ± 4.22 mg/100 g), which was 32.83% higher than the control. Previous research reported that 4–8 min of MW treatment of rapeseed could increase phytosterols by 57.73–140.96 mg/100 g in the oil extracted by cold pressing, which was 10.18–18.36% higher than the control [10,19,20]. Comparing with the MW effect on rapeseed, MW pretreatment on peanuts has more obvious improvement in the percentage of free phytosterols content enhancement in extracted oil to the initial content in the control sample.

Tocopherols are natural antioxidants that inhibit lipid oxidation in oil. There are significant differences within the tocopherols profile among different peanut cultivars. The content of four types of tocopherols (α-, γ-, δ- and β-) in peanut oils are 18–57%, 36–78%, ND-6% and ND-2%, respectively [5]. The free form of tocopherols has higher bioaccessibility than the esterified form, because it is easier to incorporate into mixed micelles [30]. The free tocopherols profile of extracted oil is shown in Table 2. β-tocopherol was not detected in the oil samples in this study. The extracted oil from untreated peanuts has 18.42 ± 0.29 mg/100 g free tocopherols including 13.87 ± 0.27 mg/100 g α-tocopherol, 3.97 γ-tocopherol and 0.58 ± 0.02 mg/100 g δ-tocopherol. MW pretreatment of peanut significantly (*p* < 0.05) increased the concentration of all types of free tocopherols in oils. With 5 min of MW treatment, the α-, γ- and δ-tocopherol in extracted oil all reached the maximum content (21.36 ± 0.24 mg/100 g, 5.66 ± 0.04 mg/100 g and 0.86 ± 0.02 mg/100 g, respectively). The total free tocopherols content in extracted oil from 5 min of MW-treated peanut was 27.88 ± 0.27 mg/100 g, which was 51.36% higher than the control. The damages in the lipoprotein membrane surrounding individual lipid bodies and the promoting passage of oil from cell membrane may contribute to the release of tocopherols and improving their content in the extracted oil. Contrary to our results, decreasing tocopherols content of oil from 6–30 min MW roasted peanut was reported [24]. The tocopherols content in extracted oil from MW-treated rapeseed increased to maximum at 6 min radiation for dehulled seeds and 4 min radiation for whole seeds, and then decreased with longer treatment time. Total tocopherols in oil from MW treated rapeseed was increased to 1.08–2.61 mg/100 g, which was 1.5–4% higher than control [19]. Similar results were reported [20]. With an initial moisture level of 13–15%, the tocopherols content in oil from treated rapeseed increased to maximum at 4–5 min MW exposure period. Total tocopherols in oil from MW treated rapeseed increased to 3.09–6.06 mg/100 g, which was 6.50–14.02% higher than the control. The lipoprotein membrane damages may contribute to the release of tocopherols and improving their content in the extracted oil. However, it can be inferred from the reported results [18,20,24] that the tocopherols could probably decompose with relatively long microwave pretreatments. It can be inferred that the tocopherols content in extracted oil from treated peanut possibly decreased with MW pretreatment longer than 5 min.

### 2.4. Effect Of Microwave Pretreatment on Oxidative Stability of Oil

**Figure 2:**
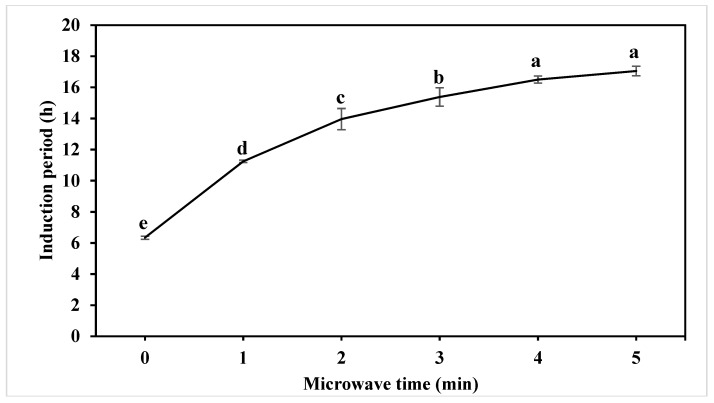
Effect of microwave pretreatment on the oxidative stability of oil. Different characters (a–e) on top of the line indicate significant (*p* < 0.05) differences among samples with different treatment times.

The oxidative stability of vegetable oil is defined as the resistance to oxidation during processing and storage [31]. The induction period (IP) is an important parameter in identifying the oxidative stability of oil. As shown in Figure 2, MW pretreatment on peanut significantly (*p* < 0.05) increases the IP of extracted oil. Oil extracted from untreated peanut has the lowest IP (6.34 ± 0.10 h). With 1, 2, 3, 4, 5 min MW pretreatment on peanut, the IP of extracted oil increased to 11.24 ± 0.08 h, 13.96 ± 0.68 h, 15.38 ± 0.59 h, 16.50 ± 0.23 h and 17.05 ± 0.31 h, respectively. These results concur with previous research [9,10,17,18,20]. The oxidative stability of vegetable oils is influenced by many factors, mainly fatty acid composition, antioxidants and minor compounds [10]. As mentioned before, the darkening of extracted oil from MW-treated peanut may be due to the formation of phospholipid non-enzymatic browning products, which are known to have strong antioxidant activity [27]. Tocopherols and phytosterols have also been reported to contribute to the increased oxidative and shelf life of vegetable oils [32,33]. Five minutes of MW treatment on peanut could inactivate 65% lipoxygenase activity and 78% lipase activity [34]. The inactivation of oxidative enzymes may possibly contribute to the higher oxidative stability of oil. With MW pretreatment on peanuts, increased tocopherols and phytosterols content, possible phospholipid non-enzymatic browning products and inactivation of oxidative enzymes are responsible for the improvement of the oxidative stability of extracted oil. Longer shelf life will provide stronger market competitiveness for oil products.

### 2.5. Effect of Microwave Pretreatment on Volatile Compounds of Oil

Flavor is the most important sensory quality of oil. Because of the strong nutty and roasty flavor, aromatic roasted oil is more popular with consumers than cold-pressed peanut oil. Pyrazines accounting for 50% relative percentage area were the highest contributors to the volatile profile of aromatic roasted peanut oil [35]. As can be seen from Table 3, most of the pyrazines showed a nutty and roasty flavor. Their contribution to the whole flavor of oil will be based on their odor threshold values. 2,5-dimethyl-pyrazine is highly correlated to a roasted peanut flavor and aroma [36]. A total of 101 volatile compounds in pretreated and untreated samples were identified by HS-SPME/GC-MS. The key pyrazine compounds in extracted oil from MW treated and untreated peanut by cold pressing re shown in Table 3. There were no pyrazines detected in the extracted oil from untreated and 1 min MW-treated peanut. With 2 min MW treatment on peanuts, 0.47 ± 0.02% trimethyl-pyrazine was detected in the volatile profile of the extracted oil. The relative content and peak area of pyrazines in extracted oil significantly (*p* < 0.05) increased in the period of 3–5 min of MW treatment. This observation was consistent with the previous research results, which found pyrazines in extracted oil significantly increased after 6 min of MW treatment on rapeseed [21]. With 4, 5 min MW treatment on peanuts, the relative content and peak area of pyrazines in extracted oil reached the maximum (33.37 ± 0.24% and 31.56 ± 0.31 × 10^7^, respectively). The relative content and peak area of 2,5-dimethyl-pyrazine in extracted oil reached the maximum (5.56 ± 0.12%, 4.34 ± 0.06 × 10^7^, respectively) with 4, 5 min MW treatment on peanuts, respectively. Pyrazines are heterocyclic nitrogen-containing compounds derived from nonenzymatic protein–sugar interactions [37]. The formation of pyrazines in peanuts required at least 30 min roasting, and could reach a temperature of about 180 °C. Forty to 50 min roasting of peanuts leads to the formation of large amounts of pyrazine compounds [34]. With 5 min of MW treatment, the peanut temperature could reach 125–130 °C [38]. Compared with roasting, MW could promote the formation of pyrazines by a shorter period of thermal treatment under a relatively low temperature, which could be considered the most suitable way to improve the flavor of cold-pressed peanut oil. The MW pretreatment solved the bottleneck of cold-pressed peanut oil in industrial application and promotion. As a high-quality vegetable oil, flavored cold-pressed peanut oil provided a new choice for consumers.

## 3. Materials and Methods

### 3.1. Materials

Peanut samples (LUHUA11, Shandong Province) were purchased from the local market (Beijing, China). Tocopherols, Sterol and Pyrazines standards were purchased from Sigma-Aldrich Co. (St. Louis, MO, USA). C6-C23 n-alkanes standards were purchased from Shanghai Chemical Reagent Co. (Shanghai, China). Chromatographic-grade methanol and acetonitrile were purchased from Thermo Fisher Scientific Inc. (Waltham, MA, USA). Other chemicals and reagents used in this study were of analytical or chromatographic grade and were purchased from Sinopharm Chemical Reagent Co., Ltd (Shanghai, China).

### 3.2. Microwave Pretreatment

For each microwave (MW) pretreatment of peanuts, 500 g of shelled peanuts were placed in a 18 cm diameter petri dish inside the microwave oven (Model: MG720KG3-NA1). The samples were pretreated at a frequency of 2450 MHz (med-high setting, 700 W) for 1–5 min with 1 min intervals. The peanut sample without MW pretreatment (0 min irradiation time) was used as the control sample. The MW-pretreated samples were cooled to room temperature for the following cold-pressing.

### 3.3. Cold Pressing

Peanut oil was obtained by hydraulic press (Model QYZ-230, Taian, China). The cold-pressing parameters were between 22–25 MPa and 60 °C inside the press temperature for 30 min. There was no further oil obtained by longer pressing time. Residue particles in the oil were removed by 4300 r/min centrifugation for 10 min. The collected oil samples were stored at 4 °C for the following experiments.

Determination of oil content in peanut and peanut meal was according to the ISO method 659 [39]. Oil extraction yield was calculated on the basis of the following formula [40]:

(1) $$Y=100\;\times \;\frac{R_{S}}{R_{c}}$$

where, Y = oil yield, R_S_ = the ratio of non-lipid components in seed to oil content in seeds, R_C_ = the ratio of non-lipid components in cake to the residual oil content in cake.

### 3.4. Physicochemical Properties and Oxidative Stability

Determination of acid value and peroxide value were according to AOCS Official Method Cd 3a-63 and Cd 8b-90, respectively [41,42]. The color measurements were using Lovibond PFXi-880/AT in a 1-in (25.4 mm) cell. The oxidative stability index (OSI) of the oil samples was determined using Rancimat (Metrohm model 743, Metrohm KEBO Lab AB, Herisau, Switzerland) according to the method described by Azadmard-Damirchi et al. (2010). Oil samples were respectively weighed (2.5 g) into the reaction vessel and heated to 110 °C with an air flow of 20 l/h. The induction period (IP) was expressed in hours (h).

### 3.5. Determination of Free Tocopherols and Phytosterols by High-Performance Liquid Chromatography (HPLC)

#### 3.5.1. Saponification of Extracted Oil Samples

The weighed oil sample (5 g) was mixed with 50 mL of 1M potassium hydroxide in 95% ethanol in a flask. Then the 5 mL 0.57 M ascorbic acid solution was added. The flasks were shaken in a water bath at 80 °C for 30 min and cooled to room temperature. Then the solution was transferred to a separating funnel. Fifty milliliters of purified water and 100 mL hexane were added into the system. The separating funnel was vigorously shaken to ensure the unsaponifiables (tocopherols and phytosterols) were fully extracted by the hexane phase. The hexane was removed at 50 °C using a vacuum rotary evaporator. The obtained residual was re-dissolved in 2 mL ethanol for HPLC analysis.

#### 3.5.2. HPLC Analysis of Free Tocopherols and Phytosterols

Quantification of free tocopherols and phytosterols was done using a 1250 Series HPLC system (Waters, Milford, CT, USA) equipped with a UV detector (2487, Waters, Milford, CT, USA) and a C18 reversed-phase column (250 × 4.6 mm; 5 μm). The injection volume and column temperature were 20 μL and 30 °C, respectively. Tocopherols were detected at 300 nm wavelength. The mobile phase was a mixture of methanol and high-purity water (98:2, *v*/*v*). The flow rate of the mobile phase was set at 1 mL·min^−1^. Phytosterols were detected at the 210 nm wavelength. The isocratic mobile phase (acetonitrile: high-purity water = 98:2, *v*/*v*) was set at a flow rate of 1.5 mL·min^−1^. Each component was quantified using an external standard method with pure standards of tocopherols and phytosterols. Waters Breeze software (Waters, Milford, CT, USA) was used to calculate the peak areas.

### 3.6. Volatile Compounds Analysis

#### 3.6.1. Headspace-Solid Phase Micro-Extraction

SPME fibre (65 μm polydimethylsiloxane/divinylbenzene (PDMS/DVB) fiber, Supelco, Bellefonte, PA, USA) was used for flavor extraction. The fiber was previously conditioned at 250 °C for 1 h before each use. Five grams of the weighed oil sample was placed into a 20 mL glass vial which was sealed with an aluminum cover and a Teflon septum. The samples were heated at 50 °C for 20 min in a thermostatic bath with a magnetic stirrer and extracted for 40 min using an auto SPME holder containing fiber. Subsequently, the fiber was injected into the gas chromatography-mass spectrometry (GC-MS) system (Shimazu QP2010 SE, Kyoto, Japan). The volatiles absorbed by the fiber were thermally desorbed in the hot injection port of the GC for 2 min at 250 °C.

#### 3.6.2. Gas Chromatography Mass Spectrometry (GC-MS) Analysis

The GC system was equipped with a DB-WAX capillary column (30 m × 0.25 mm ID, 0.25 μm film thickness) and a trace mass spectrometer (Finnigan, San Jose, CA, US). The splitless injection mode was used. The helium was used as the carrier gas at flow rate of 1 mL/min. The injector and detector temperature were set at 250 °C and 280 °C, respectively. The oven temperature was initially set at 40 °C for 3 min, then raised to 120 °C at 5 °C/min, subsequently programmed to 200 °C at 10 °C/min, and held for 5 min. Mass spectra were recorded by electron impact ionization mode (70 eV) scanning within the mass range from 35 to 500 amu. The ion source temperature was maintained at 200 °C.

#### 3.6.3. Identification

Volatiles were primarily identified by comparison of the mass spectra with data from the mass spectra NIST database. In addition, the volatiles were identified by matching the retention indices (RI) data in the literature [43] and comparing with commercial standards. Based on the series of n-alkanes (C6-C23), RI were calculated according to the following formula.

RIx = 100n + 100 (tRx − tRn)/(tRn + 1 − tRn)(2)

where, retention time (tR) of tRn < tRx < tRn + 1; n = number of atom carbon.

### 3.7. Statistical Analysis

The experiments were performed in triplicate. The least significant difference (LSD) method was used to determine the significant difference between mean values. A confidence level was set at *p* < 0.05 and the software SPSS (IBM SPSS 22.0, Chicago, IL, USA) was used for statistical analysis.

## 4. Conclusions

Peanut microwave pretreatment prior to cold pressing was effective for improving the extraction yield, micronutrients content, oxidative stability, and flavor quality of oil. Although the acid value (AV) and peroxide value (PV) of extracted oil from MW-treated peanuts were increased, the values were both far below the limit in the Codex standard for cold-pressed and virgin oils. Comparing with the untreated sample, 5 min MW pretreatment on peanuts significantly increased the oil extraction yield, phytosterols content, tocopherols content, and the induction period of the oil extracted by cold pressing. MW pretreatment on the peanut also formed the pyrazine which contributed to improving the nutty and roasty flavor of the cold-pressed oil. In conclusion, MW pretreatment is a feasible method to improve the oil extraction yield and to obtain the cold pressed peanut oil with longer shelf life and better flavor. The economics and energy requirements for the industrial-scale continuous microwave-assisted system need to be further investigated.

## Figures and Tables

**Table 1:** Effect of microwave pretreatment on physicochemical properties of oil.

MW Time (min)	Color (Red Units)	Color (Yellow Units)	Acid Value (mg KOH/g Oil)	Peroxide Value (meq O_2_/kg Oil)
0	0.00 ± 0.00 ^f^	1.20 ± 0.10 ^c^	0.31 ± 0.02 ^d^	0.54 ± 0.08 ^e^
1	0.10 ± 0.00 ^e^	1.40 ± 0.00 ^c^	0.41 ± 0.01 ^c^	0.64 ± 0.00 ^e^
2	0.20 ± 0.00 ^d^	2.37 ± 0.44 ^b^	0.43 ± 0.03 ^bc^	2.58 ± 0.08 ^d^
3	0.23 ± 0.03 ^c^	2.67 ± 0.24 ^b^	0.45 ± 0.01 ^ab^	3.86 ± 0.16 ^c^
4	0.30 ± 0.00 ^b^	2.67 ± 0.24 ^b^	0.46 ± 0.02 ^ab^	5.10 ± 0.00 ^b^
5	0.40 ± 0.00 ^a^	3.47 ± 0.38 ^a^	0.47 ± 0.00 ^a^	5.30 ± 0.08 ^a^

Values represent means ± standard deviation of triplicate tests. Values in the columns with different superscripts ^a–f^ are significantly different from each other according to least significant difference tests (*p* < 0.05).

**Table 2:** Effect of microwave pretreatment on free phytosterols and tocopherols content (mg/100 g) in oil.

MH Time (min)	Phytosterols	Tocopherols			
	Total	α-Tocopherol	γ-Tocopherol	δ-Tocopherol	Total
0	273.55 ± 2.51 ^f^	13.87 ± 0.27 ^e^	3.97 ± 0.06 ^e^	0.58 ± 0.02 ^c^	18.42 ± 0.29 ^e^
1	298.49 ± 3.26 ^e^	13.93 ± 0.17 ^e^	4.00 ± 0.07 ^e^	0.60 ± 0.04 ^c^	18.53 ± 0.22 ^e^
2	310.33 ± 5.21 ^d^	15.82 ± 0.06 ^d^	4.37 ± 0.03 ^d^	0.60 ± 0.04 ^c^	20.79 ± 0.08 ^d^
3	325.13 ± 6.76 ^c^	16.79 ± 0.22 ^c^	4.58 ± 0.08 ^c^	0.66 ± 0.01 ^b^	22.03 ± 0.25 ^c^
4	339.16 ± 3.73 ^b^	20.38 ± 0.03 ^b^	5.05 ± 0.11 ^b^	0.69 ± 0.03 ^b^	26.12 ± 0.12 ^b^
5	363.35 ± 4.22 ^a^	21.36 ± 0.24 ^a^	5.66 ± 0.04 ^a^	0.86 ± 0.02 ^a^	27.88 ± 0.27 ^a^

Values represent means ± standard deviation of triplicate tests. Values in the columns with different superscripts ^a–f^ are significantly different (*p* < 0.05).

**Table 3:** Key pyrazine compounds identified using HS-SPME and GC-MS in extracted oil from MW treated and untreated peanut by cold pressing.

Compounds	Odor Description	Microwave Pretreatment Time									
		0 min	1 min	2 min	3 min			4 min		5 min	
		Peak Area (×10^7^)	Relative Content (%)	Peak Area (×10^7^)	Relative Content (%)	Peak Area (×10^7^)	Relative Content (%)	Peak Area (×10^7^)	Relative Content (%)	Peak Area (×10^7^)	Relative Content (%)
2-methyl-pyrazine	Nutty, roasted	ND	ND	ND	1.07 ± 0.01		2.27 ± 0.02	2.92 ± 0.03	3.84 ± 0.03	3.56 ± 0.02	3.47 ± 0.01
2,5-dimethyl-pyrazine	Roasted	ND	ND	ND	2.33 ± 0.01		4.97 ± 0.03	4.23 ± 0.10	5.56 ± 0.12	4.34 ± 0.06	4.23 ± 0.07
2,6-dimethyl-pyrazine	Nutty, roasted, sweet	ND	ND	ND	0.77 ± 0.01		1.64 ± 0.03	2.07 ± 0.09	2.72 ± 0.10	2.43 ± 0.02	2.36 ± 0.03
2-ethyl-pyrazine	Peanut-butter, nutty, woody, buttery	ND	ND	ND	0.42 ± 0.01		0.90 ± 0.02	0.95 ± 0.03	1.25 ± 0.04	1.05 ± 0.04	1.02 ± 0.03
2,3-dimethyl-pyrazine	Nutty, green	ND	ND	ND	ND		ND	1.02 ± 0.01	1.35 ± 0.01	1.05 ± 0.01	1.02 ± 0.01
2-ethyl-6-methyl-pyrazine	Nutty	ND	ND	ND	ND		ND	1.18 ± 0.02	1.55 ± 0.03	1.48 ± 0.04	1.44 ± 0.03
2-ethyl-5-methyl-pyrazine	Nutty, roasted, grassy	ND	ND	ND	ND		ND	2.30 ± 0.04	3.03 ± 0.05	2.60 ± 0.02	2.53 ± 0.01
trimethyl-pyrazine	Nutty, roasted, grassy	ND	ND	0.47 ± 0.02	1.31 ± 0.06	1.44 ± 0.04	3.06 ± 0.07	2.93 ± 0.02	3.86 ± 0.03	3.34 ± 0.18	3.25 ± 0.11
2,5-dimethyl-3-ethyl-pyrazine	Nutty, roasted, earthy	ND	ND	ND	1.68 ± 0.02		3.59 ± 0.04	3.51 ± 0.11	4.61 ± 0.13	3.82 ± 0.13	3.72 ± 0.07
2,3-Dimethyl-5-ethyl-pyrazine	Nutty, roasted	ND	ND	ND	0.29 ± 0.01		0.63 ± 0.01	0.81 ± 0.04	1.07 ± 0.05	1.02 ± 0.02	0.99 ± 0.01
2-ethenyl-6-methyl-pyrazine		ND	ND	ND	0.22 ± 0.01		0.46 ± 0.02	0.33 ± 0.02	0.43 ± 0.02	0.44 ± 0.01	0.43 ± 0.01
3,5-diethyl-2-methyl-pyrazine		ND	ND	ND	ND		ND	0.81 ± 0.04	1.07 ± 0.06	1.00 ± 0.07	0.97 ± 0.04
2-methyl-6-(1-propenyl)-, (E)- pyrazine		ND	ND	ND	0.48 ± 0.02		1.02 ± 0.04	0.79 ± 0.09	1.04 ± 0.10	1.06 ± 0.05	1.04 ± 0.04
acetyl-pyrazine		ND	ND	ND	ND		ND	ND	ND	1.88 ± 0.07	1.83 ± 0.08
2-acetyl-3-methyl-pyrazine	Nutty, vegetable	ND	ND	ND	ND		ND	1.52 ± 0.08	1.99 ± 0.10	2.50 ± 0.12	2.44 ± 0.06
Total				0.47 ± 0.02	1.31 ± 0.06	8.70 ± 0.06	18.54 ± 0.13	25.37 ±0.20	33.37 ± 0.24	31.56 ± 0.31	30.74 ± 0.20

Compounds have been identified by comparison with commercial standards. Odor threshold and description in oil provided from Ref. [35]. Values represent means ± SD (n = 3). ND, not detected.
